# Requirements for Designing an Effective Metallic Nanoparticle (NP)-Boosted Radiation Therapy (RT)

**DOI:** 10.3390/cancers13133185

**Published:** 2021-06-25

**Authors:** Ioanna Tremi, Ellas Spyratou, Maria Souli, Efstathios P. Efstathopoulos, Mersini Makropoulou, Alexandros G. Georgakilas, Lembit Sihver

**Affiliations:** 1 DNA Damage Laboratory, Department of Physics, School of Applied Mathematical and Physical Sciences, Zografou Campus, National Technical University of Athens (NTUA), 15780 Athens, Greece; ioannatremi@mail.ntua.gr (I.T.); mariasouli@mail.ntua.gr (M.S.); mmakro@central.ntua.gr (M.M.); 2 2nd Department of Radiology, Medical School, National and Kapodistrian University of Athens, 11517 Athens, Greece; ellas5@central.ntua.gr (E.S.); stathise@med.uoa.gr (E.P.E.); 3 Atominstitut, Technische Universität Wien, Stadionallee 2, 1020 Vienna, Austria; 4 Department of Physics, Chalmers University of Technology, SE-412 96 Gothenburg, Sweden

**Keywords:** metallic nanoparticles (NPs), radiosensitization, radiation therapy, X-rays, ions beam therapy

## Abstract

**Simple Summary:**

Recent advances in nanotechnology gave rise to trials with various types of metallic nanoparticles (NPs) to enhance the radiosensitization of cancer cells while reducing or maintaining the normal tissue complication probability during radiation therapy. This work reviews the physical and chemical mechanisms leading to the enhancement of ionizing radiation’s detrimental effects on cells and tissues, as well as the plethora of experimental procedures to study these effects of the so-called “NPs’ radiosensitization”. The paper presents the need to a better understanding of all the phases of actions before applying metallic-based NPs in clinical practice to improve the effect of IR therapy. More physical and biological experiments especially in vivo must be performed and simulation Monte Carlo or mathematical codes based on more accurate models for all phases must be developed.

**Abstract:**

Many different tumor-targeted strategies are under development worldwide to limit the side effects and improve the effectiveness of cancer therapies. One promising method is to enhance the radiosensitization of the cancer cells while reducing or maintaining the normal tissue complication probability during radiation therapy using metallic nanoparticles (NPs). Radiotherapy with MV photons is more commonly available and applied in cancer clinics than high LET particle radiotherapy, so the addition of high-Z NPs has the potential to further increase the efficacy of photon radiotherapy in terms of NP radiosensitization. Generally, when using X-rays, mainly the inner electron shells are ionized, which creates cascades of both low and high energy Auger electrons. When using high LET particles, mainly the outer shells are ionized, which give electrons with lower energies than when using X-rays. The amount of the produced low energy electrons is higher when exposing NPs to heavy charged particles than when exposing them to X-rays. Since ions traverse the material along tracks, and therefore give rise to a much more inhomogeneous dose distributions than X-rays, there might be a need to introduce a higher number of NPs when using ions compared to when using X-rays to create enough primary and secondary electrons to get the desired dose escalations. This raises the questions of toxicity. This paper provides a review of the fundamental processes controlling the outcome of metallic NP-boosted photon beam and ion beam radiation therapy and presents some experimental procedures to study the biological effects of NPs’ radiosensitization. The overview shows the need for more systematic studies of the behavior of NPs when exposed to different kinds of ionizing radiation before applying metallic-based NPs in clinical practice to improve the effect of IR therapy.

## 1. Introduction

Many different tumor-targeted strategies are under development worldwide to limit the side effects and improve the effectiveness of cancer therapies, such as the currently developed very high dose rate FLASH therapy, chemotherapy, immunotherapy, intensity modulated radiation therapy, biology-driven personalized radiotherapy, ion beam radiotherapy, target-alpha-therapy, high intensity focused ultrasound therapy, hyperthermia, biophotonic therapy, etc. During the last 10–15 years, advances in nanotechnology also gave rise to trials with various types of metallic nanoparticles (NPs) to sensitize cancer cells to radiotherapy (RT), and was used in different medical applications ranging from contrast agents in site-specific imaging to carriers for drug, heat, and gene delivery into tumors. The radiosensitizing effect by an iodine contrast agent was first reported by Matsudaira et al. in 1980 [1], and the radiosensitization effect by high-Z metallic was first observed in patients with metallic implants who received radiotherapy for the treatment of mandibular [2] and head and neck cancers [3]. At that time, the radiation modification effects associated with metallic implants caused troubles for the medical radiation physicists, leading to the conceptualization of an appropriate dosimetry protocol by the American Association of Physicists in Medicine (AAPM) to overcome the arising problems [4]. Image reconstruction errors due to radiosensitization from metallic implants are also known affects in all the radiation therapy planning processes and is discussed in [5]. Metal complexes based of platinum demonstrated an essential impact in cancer chemotherapy. However, other high-Z metal such as titanium, ruthenium, copper and silver shown promising cytotoxic and chemotherapeutic characteristics in recent preclinical research, while iron, cobalt, and gold were already used in phase I and phase II trials [6].

Many studies [7,8] review the efficacy of metallic-based nano-agents expressing radiosensitizing and synergistic effects for radiotherapy (including gamma-rays, X-rays, and charged particles), and they termed these NPs as “NanoEnhancers”. They briefly present several categories of metallic-based NanoEnhancers for ionizing radiation (IR), as: gold (Z = 79), platinum (Z = 78), hafnium (Z = 72), gadolinium (Z = 64), and iron (Z = 26) based NPs, as well as theragnostic multimetallic nanocomposites for future RT. The enhanced synergistic effects and therapeutic outcome of high-Z metallic NPs mediated radiosensitization were tested in multiple preclinical tumor models, including glioblastoma [9]. Moreover, based on the success of preclinical studies, several clinical trials are underway to show the efficacy of the high-Z metallic NP mediated radiosensitization [9].

**Figure 1:**
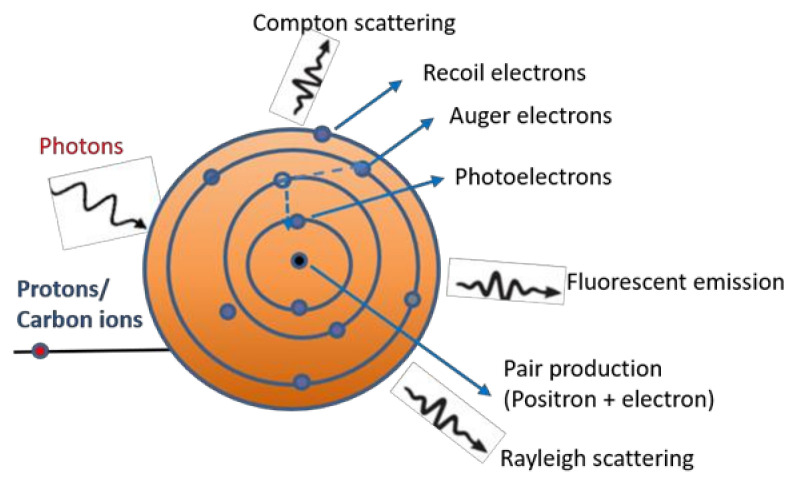
Interaction of X-rays, protons, and carbon ions with high-Z metals.

The radiosensitization by high-Z metallic NPs is caused by a physical dose enhancement followed by radiochemical and biological reactions in the tissue. The physical dose enhancement is caused by generation of secondary X-rays, photoelectrons, and Auger electrons. The chemical/biological steps include oxidative stress and reactive oxygen species (ROS) production, DNA damage and reduce repair, cell cycle arrest, and bystander effects. When using X-rays, mainly the inner electron shells are ionized, which creates cascades of both low and high energy Auger electrons. Depending on the energy of the incoming photons, protons or carbon ions, and the atomic number of the irradiated metallic NPs, Compton electrons, scattered electrons, positrons, and fluorescent emission can occur, as is schematically shown in Figure 1. During proton and carbon ion irradiation, fragmentation reactions can also occur.

For photon irradiation, the photoelectric cross-section effect depends roughly on (Z/E)^n^, with n≈3–4, where E is the energy of the incoming photon and Z is the atomic number of the target atom. The photoelectric effect is therefore dominant at lower energies and is prevailing until the photon energy reaches a medium energy (typically up to ≈500 keV) with high Z atoms (Z ≈ 60–80). Therefore, the most explored high Z radio-sensitizing agents are typically comprised of gold (Z = 79), platinum (Z = 78), hafnium (Z = 72), or gadolinium (Z = 64) [10], but even e.g., silver (Z = 47) and iron (Z = 26) were studied. Increasing the Z of the NPs is enhancing the photoelectric and Compton effects when they are exposed to X-rays. High-Z NPs are therefore more radio-sensitizing when using X-rays than low Z NPs. Gold is a promising radio-sensitizer due to its high atomic number and mass energy coefficient relative to soft tissue. In addition to that, it is very inert and highly biocompatible.

In conventional radiation therapy, X-rays consist of a mixture of x-ray waves with various energy levels. By using a monochromator, the white X-rays which are normally used in medical applications can be separated into monochromatic X-rays, with each having a single energy level. Irradiation of high Z NPs with a synchrotron producing monochromatic X-rays with an energy level that is same or higher than the K-edge energy of the metallic material used in the NP is very promising [11]. These local ejections of K-shell electrons result in the production of Auger electrons that cause DNA damage which can lead to cell killing. Most of these studies were performed at synchrotron radiation sources in ESRF France. For example, Dalzon et al. found an improved radiotherapeutic effect of synchrotron radiation when combined with iron oxide NPs [12]. Matsumoto et al. irradiated tumor spheroids with a synchrotron producing monochromatic X-rays in combination with gadolinium-loaded MCNs (mesoporous silica nanoparticles) [13]. This resulted in a complete tumor destruction at an energy level of 50.25 KeV. The same effect, however, was not observed at an energy level of 50.0 KeV, which suggests that a precisely tuned monochromatic x-ray can target a tumor while sparing neighboring cells. Bulin et al. presented LaF_3_:Ce nanoscintillators (doped lanthanum fluoride nanoparticles) as potent radiosensitizers [14]. They performed a comprehensive study with Geant4-based Monte Carlo simulations, as well as in vitro and in vivo experiments. In a syngeneic orthotopic glioblastoma in vivo model they monitored toxicity and radiotherapy enhancement in combination with monochromatic X-rays, which showed a 15% tumor remission. Finally, Gagliardi et al. used synchrotron radiation is PRESAGE dosimeters in the presence of gold and bismuth NPs to validate the dose enhancement effect. By using these NPs in different concentrations and sizes, the observed dose enhancement was highest for the 95.3 keV mean energy synchrotron beam (16–32%), followed by the 150 kVp superficial beam (12–21%), and then the 6 MV beam (2–5%) [15].

When using high LET particles, e.g., carbon ions for therapy, mainly the outer shells are ionized, which give rise to electrons with lower energies than when using X-rays. However, the amount of the produced low energy electrons is higher when exposing NPs to ions than when exposing them to X-rays. Since ions traverse the material along tracks, and therefore give rise to much more inhomogeneous dose distributions than X-rays, there might be a need to introduce a higher number of NPs when using ions compared to when using MV energies to create enough primary and secondary electrons to get the desired dose escalations. However, the use of metallic NPs in radiotherapy at clinical MV energies has the potential to increase the dose deposited in target volumes even at relatively low concentrations [16,17]. For example, dose enhancement ratios ranging from 14–287% were observed with within 6 MV Linac beams using gold nanoshells with shell thickness varying from 10–100 nm [18].

Simulation studies support that the radiation sensitization enhancement factor is higher for kV photons than photons in the MV range [19,20]. This was also experimentally verified by Chithrani et al. [21], who exposed HeLa cells with 50 nm gold NPs to photons with energies from 105 KVp to 6 MVp. Recent Monte Carlo simulations of the radio-enhancement effects of different concentrations, sizes, clustering of gold NP (AuNP) bombarded with photons, protons, and carbon ions with a wide range of energies show that in all radiation modalities, the dose enhancement increased linearly with the AuNP concentration and decrease with AuNP size and degree of clustering [22]. However, the NPs size and concentration are correlated with cellular uptake and toxicity, as it is discussed further down. The dose enhancement effect in cytoplasm and nucleus is higher for 50 keV X-rays than for 10 MeV protons and 100 MeV carbon-ions. This is directly correlated with the previously mentioned produced electron energies for each type of IR. Geant4 simulations also show that the electron spectra from X-rays at the surfaces of AuNPs show a strong distribution of Auger electrons around 2 keV, while the number of the emitted low energy electrons is about 28 times larger for 10 MeV carbon-ions in comparison with 1 MeV protons correlating with the 27 times higher LET [22]. However, in the case of proton interactions the production of radicals upon energy deposition (G-value) is sensitive to the size of the NPs with more radical interactions for AuNP with 2 nm than for particles with 50 nm diameter for the same concentrations. The dose enhancement linearly decreased with AuNP size and degree of clustering [22]. Similar ROS enhancement factors were observed when irradiating gadolinium oxide (Gd_2_O_3_) NPs with X-rays and protons with the same dose [23]. In general, ion irradiation damage is thought to be due to either nuclear or electronic energy loss [24]. In electronic stopping power, damage is believed to be produced only above a threshold, whereas nuclear damage is still significant in the energy regime just above the energy ion track threshold [25]. For ionizing particles, the damage produced by nuclear stopping power is more considerable with respect to electronic stopping power.

Nanoparticles enter the body by crossing outer layers either in skin or tissue organs, they can otherwise be inhaled, infused, or injected into the body or into the bloodstream. NPs used for radiosensitization can be either therapeutic agents (e.g., cisplatin, drug conjugated/encapsulated NPs) or inert therapeutic NPs (e.g., AuNPs) [26,27,28,29]. The first can sensitize cells alone, the second or a combination of both can act as radiosensitizers when combined with IR exposure [30,31]. NPs can be administered either locally by surgical or nonsurgical procedures along with NP/drug-loaded implants or systemically by injection, inhalation etc., depending on the selected route and the tumor site. Desired is a systemic administration of NPs (with or without anticancer drugs) to target cancerous cells at the desired tumor site with marginal loss in blood circulation. The two main targeted delivery pathways are passive targeting and active targeting [32,33]. Passive targeting uses the enhanced permeability and retention (EPR) effect, allowing macromolecules of diameter up to 400 nm to pass into the tumor cells (drug/NP complex circulates through the bloodstream and is driven to the target site by affinity or binding which are influenced by properties like pH, temperature, molecular site, and shape), whereas active targeting uses surface ligands (peptides, antibodies etc.) to enhance accumulation and cellular uptake of NPs via receptor-facilitated endocytosis [33]. Research shows that smaller NPs (~10 nm) reach most organs, while larger NPs (>100 nm) are mainly held in the spleen and liver. For example, an in vivo study in mice reported that intravenous injection of fluorescent PLGA-NPs of sizes 200 nm and 500 nm were accumulated highest in the liver, spleen, and lungs [34]. Inhalation of NPs is usually used for treatment of lung cancers, as it is more successful in terms of accumulation and retention since the escape of NPs into circulation is limited [35,36]. Some of the NPs used for delivery via inhalation include GNPs, cisplatin NPs, carboplatin NPs [36]. Monte Carlo simulations showed an increased DEF (decreasing tumor volume) when applying NPs through inhalation in combination with chemo-radiotherapy with 6 MV external beam RT [36]. On the other hand, local delivery results in high concentration of NPS in the desired area, which decreases body toxicity. This method can be very advantageous especially in tumor sites, where systemic delivery fails. This delivery system includes direct intratumoral injection, or the use of biodegradable polymeric implants such as gels, wafers, millirods etc. [37,38]. However, the discussion regarding delivery of NPs is directly correlated with their material, size, shape, surface functionalization etc.

The physicochemical properties (size, shape, coating, functionalization, etc.) of nano-agents influence their pharmacokinetics, bioavailability, biodistribution, as well as targeting and intracellular delivery. The physical and chemical properties of materials are different in the nanometer scale than in macroscale [39] and the size of the NPs used for radiosensitization affects both how they interact with the biological system and how they interact with the incoming radiation. The sizes of the NPs are a critical parameter since small NPs with sizes ~10 nm are able for nuclear uptake but they are easily cleared from circulation through kidneys. On the other hand, too large NPs ~100 nm can limit the membrane wrapping efficiency [40]. Moreover, the concentration of the NPs in combination with the size can affect the NPs efficiency and toxicity. High concentrations of some metallic NPs can increase the probability for aggregations and the toxicity, while NPs can be not toxic at low concentrations [41,42].

The surface functionalization of NPs is strongly correlated with the cellular uptake and the subcellular location. The functionalization can be achieved by coating NPs surface with polyethylene glycol (PEG) or by attaching on their surface antibodies, phospholipids, polymers and other biomolecular linkers depending on chemical moiety that is over expressed in each type of cancer cell [43,44]. The NPs should overcome several extra- and intracellular barriers to reach their target cells. Thus, the surface functionalization should be the proper one to circulate in blood plasma avoiding the activation of the reticuloendothelial system (RES) and to be able to escape from endosome after their internalization in the cells.

On a cellular level, NPs can be internalized in the nucleus, cell organelles, such as mitochondria and endoplasmic reticulum or located in the cell membrane. The subcellular location affects the radiosensitization efficacy [45,46,47]. The highest dose enhancement seems to be achieved when metallic NPs are located close to the nucleus where the energy deposition from Auger electrons is highest [48]. However, simulations show that when metallic NPs tend to localize in the cytoplasm like in mitochondria can also be effective radiosensitizers [46,49]. Hossain et al. comparing different materials of nanoparticles for a given size, concentration and location found that bismuth NPs demonstrated 1.25- and 1.29-times higher dose enhancements than Au and platinum NPs.

**Figure 2:**
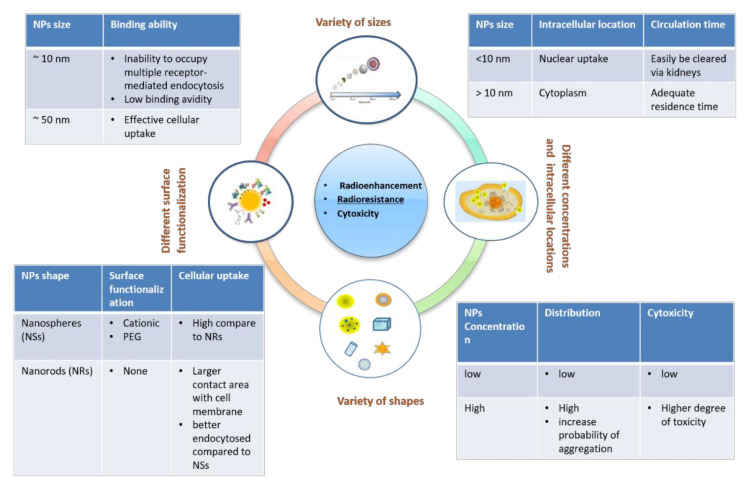
Key parameters for designing an effective NP-based radiosensitizing effect in RT, and known main mechanisms associated with use of metallic NPs in RT.

Figure 2 summarizes the key parameters for designing an effective NP-based radiosensitization, in terms of NP size, shape, functionalization, cellular uptake, body circulation and impending toxicity.

### 1.1. Most Promising Metallic NPs Proposed for RT

#### 1.1.1. Gold (Au, Z = 79)

Au is a high Z, very inert transition metallic. Owing to their unique physical, chemical, optical and electronic properties, AuNPs were exploited for a wide range of applications in diagnostics, imaging, delivery, photothermal (use of electromagnetic radiation) and ionizing RT. Multidisciplinary research performed over the past decade demonstrated the potential of AuNP-based radiosensitizers and identified possible mechanisms underlying the observed radiation enhancement effects of AuNPs [50]. The AuNP size, shape, surface coating, and functionalization can be fine tailored, which enables fine-tuning of the particle properties that is an important criterion to be used in different medical applications. AuNPs can be fabricated in different shapes/morphologies such as spheres, tubes, cubes, cages, stars hollow shells, hollow spheres, and hollow tubes. Au nanorods might have greater cellular uptake compared to spherical NPs as they can have larger contact area with the cell membrane receptors if they interact with their longitudinal axis. However, surface coating plays a key role for the cellular uptake to create efficient bonds with the receptors on the cell’s surface [51]. Recently, Li and Lane [52] summarized the aspects of size, shape and surface chemistry for fundamental understanding of how certain physicochemical parameters affect the AuNPs ability to overcome biological obstacles in vivo and reach their intended target. They focused on how AuNPs face biological obstacles that are seen by intravenously administered nanomedicines including opsonization, cellular internalization, tumor accumulation, and elimination from the body. Recently, the correlation between the sizes of the AuNPs and uptake, toxicity, radiosensitization, and radiation survival was reviewed [53,54]. This study summarizes several in vitro and in vivo experimental results on uptake and radiation therapy enhancement. Non-targeted AuNP with sizes around 50 nm seem to have maximum cellular uptake.

#### 1.1.2. Platinum (Pt, Z = 78)

Pt is a high-Z member of the platinum group of elements of the periodic table of elements. Pt has significant radiosensitizing properties and Pt anticancer agents represent a great success story which were introduced to the market almost 40 years ago. Three Pt-containing drugs are approved worldwide for treating cancer in humans, cisplatin carboplatin, and oxaliplatin. In addition, nedaplatin, lobaplatin, and heptaplatin are approved for use in specific countries. PtNPs were widely studied in biomedical applications such as drug delivery bodies [55], contrasts agents in computed tomography imaging [56], scavenger of reactive oxygen species [57], and radiosensitizers [58]. Recently, Li et al. show that ultra-small platinum NPs (1.7 nm) PtNPs with minimal inhibition concentration of 4.8 mg/L can penetrate the 150 nm membrane thickness of D. radiodurans cells and overcome its radioresistance [59] and the PtNPs enhanced the effects of 1.25 MeV gamma ray radiation by more than 40%. PtNPs were shown to possess the capability to enter the cells, generate oxygen, and cause single strand breaks (SSB) and double strand breaks (DSB) in DNA though their interaction with X-rays, gamma rays, or ions. The size of the platinum NPs seems to strongly affect their toxicokinetic. However, there are only few studies on the size dependent toxic effects. Buchtelova et al. tested the cytotoxicity of three different primary sizes of PtNPs (~10 nm, ~14 nm and ~20 nm) capped with biocompatible polymer polyvinylpyrrolidone (PVP) on prostate, breast, and neuroblastoma cancer cell lines [60]. The results showed that the smaller PtNPs exhibited the highest cytotoxicity, while the haemotoxicity seems not to be affected by the size. Porous PtNPs were proposed as a new nanomedicine platform to overcome radioresistance and to enhance radiotherapy in vivo. The combination of the high Z and the oxygen generation capability can significantly increase the X-ray radiation energy deposition within the cells, increase radiation-induce DNA damage, ROS stress and increase tumor oxygenation by converting endogenic H_2_O_2_ to O_2_ with no apparent toxicity in animals [61]. Pt nanodedrimers (PtNDs) exhibit high biocompatibility and radiosensitization toward Hela cell line up to 0.1 mM concentration in all the tested sizes of 29, 36, 42, and 52 nm diameters. Among them, the PtNDs with 36 nm in diameter demonstrated the highest sensitization Enhancement Ratio (SER) after irradiation with 6 MV photon beam. The results demonstrate PtNDs as a novel and promising radiosensitizer in radiotherapy but of possible high toxicity [62].

#### 1.1.3. Hafnium (Hf, Z = 72)

Hf is a chemically stable inert transition metallic with high atomic number and electron density. Hf dioxide/hafnia (HfO_2_) is also chemically inert and has a high dielectric constant, high melting point, high density, high refraction index, and is transparent to visible light. In addition to the mentioned properties, HfO_2_ does not have any adverse reactions in biological systems and HfO_2_ NPs have therefore promising potentials to act as radiosensitizing and X-ray contrast agents [63,64]. HfO_2_ has also photo-luminescent properties [63] which indicates that HfO_2_ NP is a multifunctional theragnostic candidate. NBTXR3 is a functionalized 50-nm-sized crystalline HfO_2_ NP, bearing a negative surface charge, developed by Nanobiotix [65]. NBTXR3 was originally designed for direct local intratumoral injections and subsequent radiosensitization. Preclinical studies showed that NBTXR3 has a physical mode of action that does not target specific biological pathways, and might provide an opportunity to improve patient outcomes in many types of cancer [66,67]. The results of the Act.In.Sarc study [66] suggest that NBTXR3 activated by radiotherapy could represent a new treatment option in patients with e.g., locally advanced soft-tissue sarcoma of the extremities or trunk wall. Moreover, these data open a large field of applications and justify ongoing studies evaluating NBTXR3, including phase 1–2 trials in head and neck squamous cell carcinoma, liver cancer, prostate cancer, rectal cancer, and recurrent or metastatic head and neck squamous cell carcinoma or metastatic non-small-cell lung cancer [66]. Since NBTXR3 improves the efficacy of radiotherapy, all patients with resectable tumors eligible for preoperative radiotherapy treatment could benefit from its use [66].

#### 1.1.4. Gadolinium (Gd, Z = 64)

Gd is the most widely used paramagnetic element for MRI-positive contrast agents, favored for its seven unpaired electrons and relatively long electronic relaxation times. Gd chelates is commonly used as an MRI-contrast agent and for more precise and accurate irradiation in MRI-guided radiotherapy. Several large studies demonstrated that Motexafin Gd (MGd), which is a metallotexaphyrin that can catalyse the oxidation of intracellular reducing metabolites and generate ROS, is capable of enhancing the cytotoxic effects of radiation through several mechanisms as well as selectively inhibiting tumor cell growth by itself [68]. Consequently, Gd-based agents show great promise for multifunctional theragnostic (diagnostic and therapeutic) applications in clinical practice [63].

#### 1.1.5. Silver (Ag, Z = 47)

Although Ag is a transition metallic with a lower atomic number than Au, it has promising properties as a radiosensitizer. AgNPs are less inert and biocompatible than AuNPs [29,69], but AgNPs are attractive in biomedicine due to that they are cheaper and they have unique physicochemical properties. In recent years, the anticancer effect of AgNPs was widely studied both in vitro and in vivo, for e.g., cervical cancer, breast cancer, lung cancer, hepatocellular carcinoma, nasopharyngeal carcinoma, hepatocellular carcinoma, glioblastoma, colorectal adenocarcinoma, and prostate carcinoma [70]. Different surface functionalization, shapes and sizes of AgNPs were studied for their effectiveness in cancer treatment combined with IR [29]. In [70] different AgNPs synthesis methods, including physical, chemical and biological procedures, as well as medical applications and biosafety are reviewed. AgNPs were used for antimicrobial and cancer therapy, as well as for promotion of wound repair, bone healing, and as vaccine adjuvant, anti-diabetic agent and biosensors. In RT, the anti-proliferative effects of AgNPs might result from different underlying mechanisms compared to when using AuNPs [63,70]. It was suggested that induction of apoptosis, production of ROS, inhibitory action on the efflux activity of drug-resistant cells, and reactivity with glutathione (GSH) molecules are involved in the radiation enhancement effect.

#### 1.1.6. Iron (Fe, Z = 26)

Fe belongs to the first transition series of the periodic table and is by mass the most common element on Earth. Fe-based NPs were investigated as theranostic magnetic NPs [71,72], including inorganic paramagnetic iron oxide (or magnetite) NPs, or superparamagnetic iron oxide NPs (SPIONs) [73]. Fe oxide NPs (IONs) were used as negative T2 MRI contrast agents and they are considered ideal agents for diagnosis, treatment, and treatment monitoring of cancers because of their excellent properties, such as facile synthesis, biocompatibility, and biodegradability [63]. IONs have therefore potential applications not only as MRI contrast agents but also in photothermal therapy (PTT), photodynamic therapy (PDT), magnetic hyperthermia, and chemo/biotherapeutics [63].

## 2. Effects of Size, Shapes, and Surface Treatments

It is difficult to determine the optimum size and shape for the best radiation enhancement of NPs, since the NPs interacts both with the cell surfaces and with the different receptors, which lead to different pathways. Small NPs with sizes 1–5 nm are good for nuclear uptake, which is very important for NP-mediated radiation enhancement. Huo et al. [74] showed that in MCF-7 breast cancer cells, NPs with diameters smaller than 10 nm (2 nm and 6 nm) could enter the nucleus, whereas ones with diameter larger than 10 nm were only located in the cytoplasm. However, too small NPs cannot enhance the radiation because of their inability to occupy multiple receptor-mediated endocytosis binding sites and their low binding avidity. On the other hand, too large NPs seem too large for membrane wrapping [75], so sizes near 50 nm seem to be the optimal size for effective uptake [75,76,77].

Spherical NPs, especially the ones coated with polyethylene glycol (PEG), have higher uptake rates than other shapes [51,78,79], but other groups claim that rod-shaped NPs are better endocytosed compared to spherical ones [79,80]. In case of spherical NPs, the membrane wrapping time is smaller compared to the larger rod-shaped NPs. Herd et al. [81] investigated the uptake of three silica NPs, spherical, cylindrical and worm-like and their experiments suggest that microcytosis (clathrin-mediated endocytosis) is the most favorable mechanism for spherical NPs, whereas their worm-like counterparts underwent macropinocytosis or phagocytosis. For more details on shape and cellular uptake, see also [82]. The cellular uptake of NPs is also dependent on the surface charge since the electrostatic interactions between NPs and the cell membranes are of great importance. Cationic NPs show greater cellular uptake because a positive charge has a much more disrupted effect on the lipid bilayer [83]. Cho et al. showed that half of the cationic AuNPs diffused into cells by generating holes or disrupted the cell membranes. Some of these may refer to nonendocytosis pathways [84]. The surfaces of NPs can also be functionalized with biomolecular chains, such as peptides, PEG moieties, or Bovine serum albumin (BSA), which enhances the targeted effect of NPs especially for the nucleus [85,86,87]. NPs can also form aggregates due to exposure to a high concentration of ions when they come into contact with biological media [51]. Aggregation of NPs can quantitatively affect cellular uptake and/or cellular distribution based on the NPs’ physiochemical properties and the cell type. The most common coating for NPs, especially for AuNPs, is the citrate coating. However, the physiological pH citrate-coated AuNPs have a high zeta potential which makes particles easily to aggregate [88]. PEG coated NPs with sizes 5–60 nm on the other hand (especially AuNPs), increases stability and biocompatibility and prevents particle aggregation [78].

## 3. Biological/Biochemical Effects

Metallic-based NPs are chosen with respect to biocompatibility, low cytotoxicity, or for synergistic. The radiosensitization effects of metallic NPs can be categorized in three groups: the physical effects, the chemical effects and the biological effects. The physical dose enhancement leads to chemical effects (e.g., oxidative stress), which consequently lead to biological effects. Biological effects refer to cellular damage and include DNA damage and/or DNA repair inhibition, cell cycle effects, and cell death. Radiosensitization directly depends on the cellular and subcellular distribution of the NPs, which may damage specific cellular components such as the cell membrane, cytoplasm, nucleus, mitochondria, and endoplasmic reticulum (ER), as well as other organelles [46,49,89]. Sometimes theoretical models differ from experimental studies, which suggests that we still do not completely understand the biologically driven processes [90]. This confirms also that the physical effect alone (e.g., radiation type, NP concentration) is not responsible for the overall radiosensitizing effect. A summary of important aspects of the biological/biochemical mechanisms of NPs radiosensitization is presented in the paper by Rosa, Connolly et al. [63,91,92].

### 3.1. Nanotoxicity

The toxicity of the metallic NPs remains a great challenge to overcome in the cancer nanotechnology and is perhaps one of the main reasons for the limited number of clinical trials with NPs. Nanotoxicity depends on multiple factors such as the size, surface area, shape, concentration, surface coating, and charge of the NPs. Size dependent cytotoxicity was confirmed in vitro as well in vivo studies. Coradeghini et al. monitored the cellular uptake and toxicity of 5 nm and 15 nm AuNPs in different concentrations and incubation times using TEM and colony forming assay and found that only 5 nm NPs were toxic after 72 h at a concentration higher than 50 mM [41]. Yamagishi et al. studied the toxicity of subnanosized Pt particles with diameter less than 1 nm administered to BALB/c mice. Inflammation and hepatocyte death were observed for Pt doses higher than 15 mg/kg. However, nephrotoxicity and disruption of kidney functions were not observed in mice which were exposed to 8 nm PtNPs [93]. Pelka et al. also observed that there is an increase in toxicity with the decrease in diameter between <20 nm, <100 nm, and >100 nm particles in vitro human colon carcinoma cell line (HT29) [94]. Smaller size implies greater surface area and area to volume ratio per given mass and this may increase the biological reactivity and consequently the level of toxicity. Zhang et al. observed that low concentrations (<75 μg/mL) of very inert spherical AuNPs with an average diameter of about 15 nm do not affect the cell viability obviously and have no obvious cytotoxicity [95]. However, concentrations of AuNPs, with the same average particle size, above 150 mg/mL are toxic and lead to a decrease in cell viability. Conor et al. studied cells treated with 25 mM of citrate-capped AuNP spheres with an average diameter of 18 nm with Transmission Electron Microcopy (TEM). No difference in growth rates compared to untreated cells over the course of 5 days was observed [96].

Nanotoxicity is closely associated with NPs biokinetics, cell uptake, NPs coating agent and surface charge. Carboxyl coated Iron NPs AgNPs show greater toxicity compared to amine coated [97]. Uncoated Ag NPs were found were to be more toxic than coated ones [98]. In vivo studies demonstrated that PEG-coated 13 nm Au NPs injected to BALB/C mice induce inflammation and apoptosis in the liver and spleen [99]. The positive surface charge of NPs enhances the probability of cellular uptake in combination with the negative charged plasma membrane [100].

### 3.2. Cell Uptake and Localization

The radiosensitizing effect of metallic NPs depends strongly on the uptake by the cells, as well as on their intracellular distribution. There are many factors that influence the uptake of a NP, such as material, size, shape, surface charge, functionality some of which are described below [101,102]. NPs can enter the cell interior through energy-independent process such as simple diffusion, however most commonly NPs enter the cell membrane through the energy-dependent process, endocytosis. Through endocytosis the NPs from the extracellular environment enter the cell interior by vesicles that are generated from the cell membrane [103]. The cellular uptake route is very important for the NPs, since it determines the fate of the NPs, their lifespan and circulation inside the cell and their ability to reach certain organelles for targeted purposes or for drug releases. Endocytosis is mainly divided into two categories, phagocytosis and macropinocytosis. Phagocytosis is responsible for the uptake of vesicles with size in the μm range. Pinocytosis involves the uptake of fluids containing particles by vesicles of smaller size than those generated during phagocytosis. Pinocytosis is subdivided into macropinocytosis and micropinocytosis, with the latter being responsible for NP uptake. Micropinocytosis is also referred to as receptor-mediated endocytosis (RME). In most cases, NPs including metallic NPs are accumulated in the cytoplasm and are localized in vesicles such as endosomes and/or lysosomes or in the endoplasmic reticulum (ER) and are rarely seen inside the nucleus [104,105,106,107]. This sometimes creates conflict with those who associate NP radiosensitization with DNA damage. Peckys et al. used liquid scanning TEM to monitor 30 nm gold NPs uptake, which after 24 h incubation accumulated in these vesicles and formed clusters [108]. Ma et al. also used AuNPs and noticed that the internalized AuNPs eventually accumulated in lysosomes and caused impairment of lysosome degradation capacity through alkalinization of lysosomal pH. They also noticed that AuNPs increase autophagosome accumulation [104].

### 3.3. Oxidative Stress

Radiation causes cellular damage through direct and indirect mechanisms. Oxidative stress post IR refers to the excess production of reactive oxygen species (ROS) due to radiolysis of water molecules. These are oxygen-containing radicals and nonradical derivatives of oxygen. The main radical species are hydroxyl radical (•OH), singlet oxygen (O_2_), hydroxyl peroxide (H_2_O_2_), and superoxide (O^−2^), with the first two being the most reactive and damaging to the cells. In physiological environments, ROS are generated from oxygen mainly in mitochondria and serve as cell signaling molecules for normal biological processes. However, increased levels of ROS can cause severe cellular damage, oxidative stress, DNA damage, and lipid peroxidation [109,110]. This is why ROS are an important contributor to cellular damage of sparsely ionizing sources, such as X-rays and electrons, [111]. It is therefore very important to understand the link between the properties of metallic NPs and the intracellular generation of ROS when the NPs are exposed to IR. Radiation-induced damages and increased levels of ROS generation within cells can alter the antioxidant balance causing severe damage or cell death. NPs are located primarily in the cytoplasm inside vesicles and not very often near the nucleus. Radiation-induced ROS from irradiation of NPs do not extend far from the surrounding area, which makes it necessary to understand the local biochemical effects caused by the created ROS. The Synergistic effects of metallic NPs with IR was found in tumor cells, mitochondria, and other organelles which proves the idea of a complex cellular damage [112,113]. Mitochondrial (MtDNA) DNA damage are one the things that can trigger cell death such as apoptosis. Taggart et al. used AuNPs and identified radiosensitizing effects involving mitochondria. In these studies, they used a low energy ultrasoft X-ray microbeam (278 eV carbon K-shell X-rays) in combination with AuNPs and no nuclear DNA damage was detected, which led them to the conclusion that the radiosensitization was mostly driven by mitochondrial damage [114,115]. SUM159 triple-negative breast cancer (TNBC) cells in mice showed sensitizing effects through oxidative stress when irradiating mice with X-rays in combination with AU-TiO_2_ dumbbell-like NPs (~70 nm) [116]. In addition, AuNPs induction of ROS was seen to play again an important role in the radiosensitization of human U251 glioma cells with AgNPs (15 nm). AgNPs were found also to induce high levels of oxidative stress in TNBC (Triple-negative Breast Cancer) cells, which are more vulnerable to reagents that evoke oxidative stress compared to that of the non TNBC cells [117]. Magnetic Fe-oxide NPs (IONPs, 10 nm) functionalized with the antibody cetuximab, were found to sensitize EGFRvIII-overexpressing glioblastoma (GBM) cancer cells, whereas ROS production was involved [118]. Gd oxide 3 nm NPs in non-small cell carcinoma cell lines, irradiated with carbon ions showed increased cell death, related to oxidative stress [52].

### 3.4. Effect on Cell Cycle

In eukaryotes cells that contain nucleus, the cell cycle consists of four phases G1, S, G2 and M. For most mammalian cells growing in culture the S phase is usually in the range of 6–8 h, the M phase less than an hour, G2 is in the range of 2–4 h and G1 is 1–8 h, making the total cell cycle of the order of 10–24 h, depending on the cell type. Mother cells grow and copy their DNA and then split into two daughter cells. G1 phase is the first gap, where the cell grows and prepare for later phase. S phase is the synthesis phase where the DNA makes a complete copy (DNA replication) and duplicates the centrosome, a microtubule structure, which helps separate DNA during M phase. G2 phase is the second gap, the cell grows further, makes proteins and organelles and prepare for later phase. Finally, M phase is the Mitotic phase during which the cell divides its copied DNA and cytoplasm into two daughter cells. In this phase DNA condenses into chromosomes which are separated by a microtubules structure called mitotic spindle. Throughout the cycle, the cell has to check for any present damage, and more importantly, DNA damage. To do this, there are checkpoints between the previously described cell cycle phases. First, the G1 checkpoint checks for any DNA damage and ensures environmental conditions. If all is well, the cell progresses to the S phase, if not, it goes into cell cycle arrest (G0 phase). Second, the G2/M checkpoint, which happens in the end of G2 phase, right before Mitosis, is monitoring cell size and DNA replication. It checks whether the DNA is intact and properly replicated. If not, it goes into cell cycle arrest to perform DNA repair. IR is in some cases found to be responsible for cell cycle arrest by activating G1 and G2/M checkpoints, as it induces DNA damage, which the cell has to repair afterwards [119,120]. According to the cell cycle phase during IR, cells express different levels of radiosensitization. More specifically, cells in late G2/M phase are the most radiosensitive, following G1, early S and late S [121]. Therefore, the use of metallic NPs especially in G2/M cells can enhance the radiotherapeutic effects on cancer cells.

Several NPs such as Au, Ag, Gd, TiO_2_, and Fe oxide, were shown to radiosensitize cells by inducing cell cycle arrest. AuNPs with sizes ranging from 5 to 50 nm, used in several cancer cell lines (e.g., HEPG2, DU-145, A375, MDA-MB-231), lead to increased cell death, growth inhibition and in general in a DEF between 1.2 and 1.8, depending on size, surface modification, cell line and type and dose of IR [122,123,124,125]. Cells were irradiated with neutron (Average energy: 9.8 MeV, LET: 30–40 KeV/μm)/^137^Cs 5Gy/γ-irradiation and 5GyE/neutrons, 2Gy/^137^Cs, 4Gy/ 6-MV X-ray, respectively. In the case of HEPG2 cells, the effects produced by neutrons were found to be more pronounced than with X-rays when combining IR with GNPs. The combination of Glu-NPs (glucose-capped NPs) with IR in DU-145 inhibited cell proliferation by 26.8%, in contrast to IR alone (7.4%). Again, in MDA-MB-231 cells, compared to the inhibition with IR alone (7.07), the combination of IR with Glu-NPs inhibited cell proliferation by 22.55% and 32.14% for 16 nm and 45 nm size, respectively. On the other hand, functionalized Fe oxide NPs (10 nm) used in WEHI-164 (fibrosarcoma) cells and MTiO_2_ (mesoporous TiO_2_ NPs (45 nm)) used in 4T1-Luc (mouse carcinoma) cells, irradiated with ^60^Co γ-teletherapy Irradiator and X-rays respectively, showed cell proliferation inhibition [52,126,127]. More specifically, the irradiation of cells with 2Gy with the presence of Fe oxide NPs, resulted in 34–57% proliferation in HT1080 cells and 60–88% in WEHI-164 cells in respect to IR alone (88% and 96% respectively for each cell line). Finally, in 4T1-Luc cells x-ray irradiation in the presence of MTiO_2_ peptide-conjugated NPs effectively inhibited cell migration, as seen by cell migration and invasion assay (clonogenic assay). Gadolinium oxide NPs used in A549, NH1299, and NH1650 cells (all non-small cell carcinoma cells), irradiated with 2Gy carbon ions, resulted in a DEF of 1.1–1.2 [52]. Cell cycle arrest was observed more in the NH1650 cells.

Cell cycle inhibition is in addition to the NPs, dependent on the LET of the radiation. Cells are most radiosensitive in the M and G2 phases, and most radioresistant in the late S phase, as described above. Typical LET values at the tumor region for the different types of IR are the following: 2 KeV/μm for 250 kVp X-rays, 0.3 KeV/μm for Cobalt-60 γ-rays, 0.3 KeV/μm for 3 MeV X-rays, 12 KeV/μm for 14 MeV neutrons and 100–200 KeV/μm for heavy charged particles. Data show that at the same doses, high-LET irradiation is a more potent inducer of cell cycle delays than low-LET irradiation [128]. In general, more pronounced delays in S and G2 phase were observed with increasing LET. The role of cell cycle regulatory proteins is such that TP53 is controversial between high- and low-LET. Extensive delays in the G2 phase of cell populations were reported to occur comparing high-LET (up to 300 KeV/μm) and X-rays [129,130]. A recent study agrees with these effects and illustrate that breast cancer cells were arrested in the G2/M phase after irradiation with X-rays or carbon ions, and a more obvious increase in the G2/M phase ratio was observed in both cell lines after irradiation with carbon ions compared with X-rays at the same dose [131]. In glioblastoma and normal human fibroblasts, an increasing G2 phase arrest with LET (carbon ions 20–105 Kev/μm) was also confirmed [132]. Experiments on human fibroblasts showed differences between wildtype and TP53 mutant cells after x-irradiation, but these differences disappeared with increasing LET [128]. Exposing lymphoblastoid cells to nitrogen (140 KeV/μm) and iron ions (1,000 KeV/μm) gave a pronounced G2 phase delay and a greater inhibition of S phase compared to X-rays [133,134]. When compared a-particles with γ-radiation G2 phase arrested cells were similar for both types of IR, but G1 arrest was reduced in a-particles compared to γ-rays [135]. Exposure to protons ions (7 to 28 keV/μm) revealed a G2 arrest and for low doses (1 Gy), a G1 arrest after both proton and X-irradiation [136]. In contrast to high-LET irradiation, 3T3 fibroblast cells irradiated with soft X-rays (dose rate: 550 μGy/hr), showed that the highest percentage of irradiated cells were in G0/G1 phase compared to the control cells [137]. Results of this study suggest that low-dose soft x-ray radiation might cause an initial pause, followed by a significant increase, in proliferation. Another study which used X-rays with Hela cells showed that proliferation ability of cells irradiated with X-rays decreased with increasing doses. Within 24 h following irradiation, the S-phase of cells with different doses were decreased gradually and within 30 h following irradiation, the S phase exhibited a gradual increase. As for G1 phase, the higher the irradiation dose received, the greater the tendency of G1 phase reduction. Correspondingly, increased dose of irradiation resulted in a greater G2/M delay. Cells exposed to 4, 6, and 8 Gy returned to a normal cell cycle within 36 h, and cells exposed to >10 Gy did not return to normal levels within 48 h after IR [138].

### 3.5. Effect on DNA Damage

DNA damage plays a key role in cancer treatment. Irradiation alone can lead to a significant amount of DNA damage, such as SSBs and DSBs. However, the combination of metallic NPs with IR increases the severity and complexity of DNA damages. As previously mentioned, the physical stage includes both direct and indirect radiation actions and the use of metallic NPs can increase the amount of indirect actions of IR through the produced low energy secondary electrons, which leads to an increased ROS production. However, since NPs are rarely seen inside the nucleus and are more often trapped in cytoplasmic vesicles or in the ER, ROS do not extend far from the surrounding area and may not reach the DNA inside the nucleus. That is why early DNA damage (up to 1 h post IR) is attributed to NPs that have entered the nucleus or to these located near the perinuclear region, whereas late DNA damage is related to indirect effects [139]. Studies with gel electrophoresis on plasmids showed that metallic NPs can increase the number of initial DSBs [139,140]. Studies on AuNPs showed an increased radiosensitization due to DNA Damage. MDA-MB-231 breast cancer cells were sensitized by 2 nm AuNPs through the induction of DNA damage [141]. Immunofluorescence assays after exposure to X-rays in combination with AuNPs, quantified γ-H2AX and 53BP1 foci and lead to the conclusion that AuNPs have an impact on DNA repair [77,89]. Others do not report any influence on DNA repair due to the use of AuNPs [77,142,143]. Another study on Gd-based NPs suggest that NPs sensitize the cell by amplifying the radiation effects only in the cytoplasm and not in the nucleus, since NPs are not located inside the nucleus [144]. Glioblastoma cells were irradiated with micelles loaded with gold and superparamagnetic Fe oxide NPs (SPIONS), which lead to an ∼2-fold increase in density of double-stranded DNA breaks after γh2ax detection [145]. Many times, combined radiation treatment with different sizes of AuNPs (50 nm, 5 nm, 18 nm) and specific drugs like doxorubicin and cisplatin is very effective, leading to high levels of DNA damage and apoptosis in cancer cells (e.g., glioblastoma, MCF-7) [146,147,148]. ZnO NPs (7 nm) reportedly sensitized SKLC-6 lung cancer cells by inducing DNA damage; however, they did not sensitize MRC-5 normal lung cells [149]. This also leads to the conclusion that depending on the histological area NP radiosensitization vary. Another factor that also alters the sensitizing effect is the size and shape of NPs. In HepG2 cells DNA damage and chromosomal breaks was correlated to small NPs (5 nm), and not to larger ones (20 nm) [150]. Also, in HepG2 cells, Multiwalled Carbon Nanotubes (MWCNTs) (10–30 μm/8–15 nm, 0.5–2 μm/8–15 nm) induced single-strand DNA damage, where MWCNTs (10–30 μm/20–30 nm) did not [151]. Finally, AuNPs (3.1 nm) with positive charge induce the highest amount of damage compared to that of the negative ones [152].

### 3.6. Effect on Apoptosis, Autophagy and Senescence

Apoptosis is a type of programmed cell death, which is mediated by caspases, and can be stimulated by internal or external factors such as pathological factors, oxidative stress, death receptor proteins, chemotherapy, chemical exposure, and radiation. Apoptosis is divided in two categories: extrinsic and intrinsic. Intrinsic apoptosis is triggered by mechanisms related to the endoplasmic reticulum or mitochondria [153]. Autophagy refers to a heterogeneous group of cells signaling pathways, which enables eukaryotic cells to deliver cytosolic components to the autophagosomes-lysosomes for degradation and to recycle nutrients. Autophagy is usually stimulated by hypoxia, chemotherapy, or growth factor deprivation, and plays an important role in mediating cell death [154]. NP uptake happens primarily in the cytoplasm and as previously mentioned, NPs are many times located inside lysosomes or autophagosomes. Many studies imply that NP radiosensitization is accompanied with autophagosome accumulation, which may possibly lead to autophagic cell death [104]. However, in the past, studies indicated that NPs were not efficient to activate autophagy due to cellular defense mechanisms against NP-induced stress. Until now, it is not clear how NP radiosensitization leads to cell death and the detailed biological mechanisms are not well understood. Liang et al. reported that AuNPs in a size-dependent manor could block autophagy through lysosomal impairment leading to autophagosome accumulation [104]. Also, the use of AgNPs in glioma cells inhibited autophagy leading to increased levels of ROS and apoptosis [155]. Several studies show that AuNPs in combination with IR induce higher levels of apoptosis in cancer cells [143,156]. The size of NPs plays also an important role in apoptosis. Apoptosis rate on Lovo cells (human colon carcinoma) induced by AgNPs (10, 20, 40, 60 and 100 nm) was found negatively correlated with particle size [157]. The same was observed in HepG2 cells incubated with silica NPs (19, 43, 68 nm) [158]. U251 cells showed high levels of apoptosis after radiosensitization with 15 nm Citrate AgNPs [159]. Finally, after radiation, surviving cells may become resistance and regrow, but also some cells may undergo senescence, the so-called therapy induced senescence. These cells will undergo cell cycle arrest and their cell divisions are cessanated. The cell cycle arrest for normal somatic cells that undergo senescence is irreversible. However, the cell cycle arrest for cells that undergo radiation induced senescence is reversible and these cells can reenter the cell cycle again [160], which is essential for an efficient and successful radiotherapy. One of the main processes of action for NPs is the ROS production and cellular senescence mediated after ROS production [161] is of great importance and should be further studied.

## 4. Exp. Procedures to Study the Biological Effects of NPs’ Radiosensitization

Below, some methods to determine the radiosensitization effects of NPs after radiation are described. A number of different experiments is needed to get a holistic approach and to extract all the possible outcomes of radiosensitization based on DNA Damage, oxidative stress, cell survival (e.g., apoptosis, autophagy), cellular senescence, and signaling.

1. Transmission electron microscopy (TEM): TEM can be used to monitor the cellular uptake of NPs and it can be used to detect DNA damage after IR in the presence and absence of NPs. Sample preparations for TEM analysis consists of several stages. After IR, the basic steps for cell or tissue preparation are the following: fixation in aldehyde buffer solution, embedding in gelatin, post fixation in osmium tetroxide, dehydration in ethanol, and embedding in resin (epoxy or acrylic). The choice of fixative and resin depends on the aim of the study (ultrastructural or Immunocyto-histochemical). The chemicals used for these stages such as the aldehyde solution for fixation are adequate to simultaneously preserve the ultrastructure morphology of cell organelles and the antigenicity. However, all chemical preparation stages, if not applied correctly, may lead to image artifacts. For a more detailed information, the reader can resort to a recent technical note about all the technicalities regarding TEM [162]. There exist several other techniques to study DNA damage, e.g., Immunocytochemistry and Gel Electrophoresis, which are considerably more timesaving, require fewer demanding skills and protocols, and are less expensive. However, for detection of type and location of the DNA damage, TEM is unique since the details of the damage cannot be detected in such magnification and resolution (nm scale) with any other method. One disadvantage with TEM is the difficulty in quantifying the damage. Usually, only 10–50 cells are analyzed each time, so the method provides better quality than other methods but cannot accurately quantify the results. However, the use of TEM was performed for the detection of both single and double staining to detect clustered DNA damage and especially DSBs after IR [163,164]. TEM uses normally the immunogold-labelling technique to characterize the DNA damage. Primary antibodies target specific repair proteins are localized by secondary Au-conjugated antibodies, similar to immunocytochemistry methodology. TEM was not used to study DNA damage induced NP radiosensitization until now, but it was used to monitor cellular uptake and distribution of NPs [77,105,165]. Moreover, recently, the technical use of TEM was thoroughly described as a means of studying NP-induced radiosensitization in vitro [162].
2. Flow cytometry (FC): Flow cytometry can be used for DNA damage detection and cell cycle analysis. Propidium iodide is the most commonly used dye to quantitatively assess DNA content, and it is a very useful technique to study different checkpoints throughout the cell cycle [119]. Though flow cytometry is broadly used in radiation experiments, cell cycle analysis related to DNA damage-induced NPs radiosensitization was not performed until now. For instance, G2/M checkpoint prevents cells with damaged DNA from entering Mitosis. Phosphorylation at Ser10 of histone H3 is tightly correlated with chromosome condensation during mitosis [166]. An antibody that specifically recognizes the phosphorylated form of histone H3 (p-histone H3 Ser10) is used to identify mitotic cells. Cells are co-stained with anti-p-histone H3 Ser 10 antibody and propidium iodine to distinguish mitotic cells from G2 cells [167]. Milner et al., used flow cytometry on CHO cells to access DNA damage after irradiation with ^60^Co γ-source [168]. Nucleotides were extracted from cells, then they were stained with the fluorescent dye ethidium bromide and then exposed to laser light within FC. Flow cytometry can be also used to monitor NP uptake. For example, Shapero et al. [169] used flow cytometry to investigate cellular uptake and final localization of silica NPs of different sizes (50, 100, and 300 nm) inside A549 cells as a function of time. They showed that the uptake rate of silica NPs decreases with size; however, due to fluorescent intensity of NPs they suggested that results might be misleading if are not normalized.
3. Immunofluorescence: Immunofluorescence uses primary antibodies, labelled with fluorescent secondary antibodies for visualization, specific to targeted DNA repair enzymes. The most common target for DNA damage detection, such as DSBs, is the phosphorylated histone γ-H2AX (phosphorylation at serine 139). Foci represent the DSBs in a 1:1 manner and are used as a DNA damage biomarker. Other DNA repair markers include RAD51, 53BP1 (p53-binding protein 1), phospho-p53, and PARP1 [170]. However, the specificity whether such DSB markers recognize only DSBs is a controversial issue. γH2AX also appears in SSB sites [171]. Beside repair enzymes, primary antibodies can be used to label the damage itself. In this case, the antibodies bind to specific DNA lesions such as 8-oxo-dG, which is used to detect base lesions [172]. Recently electrophoresis-based DNA fractionation methods were used to quantify DNA damage. Detection and quantification of γ-H2AX and 53BP1 is very often employed to assess metallic NP-induced radiosensitization [77,173].
4. Agarose Gel Electrophoresis (AGE): DNA lesions can be identified through gel electrophoresis. This is a fast method that quantifies the average density of breaks and a variety of DNA lesions in nanogram quantities. AGE can be divided into two main groups: 1) Alkaline Gel Electrophoresis and 2) Glyoxal gel electrophoresis. Agarose gels can separate a mixture of molecules such as DNA fragments between 50 bp (3% agarose) and 500.000 bp (0.1% agarose) in an agarose matrix, with suitable electrophoresis buffers [174]. The gels are stained with ethidium bromide and the image acquisition of the DNA migration is performed with UV light. Alkaline agarose gels are mostly used for single stranded DNA but it is also used for others alkali-labile lesions as well such as 8-oxoguanine base lesions [175]. Glyoxal agarose gel electrophoresis fractionizes DNA the same way, but it also keeps alkali-labile sites intact.
5. Pulsed-field gel electrophoresis (PFGE) and Comet Assay (CA): Methods such as the comet assay and pulsed-field gel electrophoresis (PFGE) [176,177] are based on the detection of DNA fragments by electrophoresis. In comet assay, the cells are embedded in agarose gel on a glass slide for microscopy and the DNA fragments are fractionated by electrophoresis. Because of the tail-like images, this method is called comet assay. In PFGE, cells are embedded in agarose gel, called plugs, and then the cells are lysed in these agarose plugs. Only the DNA fragments such as DSBs migrate in the gel, while the undamaged DNA remain. The CA does not need a large number of cells for the analysis, and it is not expensive, but it cannot distinguish the lengths of the DNA fragments. PFGE is there for better to be used for quantitative purposes. Gel electrophoresis was already used in several studies concerning metallic NP (especially AuNPs) radiosensitization [178,179].
6. Clonogenic Survival Assay (CSA): One gold standard technique to access cell death and survival rate in radiobiology is CSA. After a stress induced situation, such as IR, survival assay determines the ability of cells to proliferate and form colonies after a few days’ incubation. Cells are seeded into petri dishes, treated with NPs, irradiated and then replated in low seeding densities and left to grow colonies for a few days (depending on the cell line). After these days, the colonies are stained with crystal violet in 80–100% methanol [180]. The individual colonies are counted and then a survival curve can be defined as a relationship between the radiation dose and the fraction of cells that were able to replicate and form colonies. Clonogenic assay is almost always used to compare cell radiosensitization in the presence and absence of metallic NPs [26,179,181].
7. TUNEL Assay: TUNEL (terminal deoxynucleotidyl transferase dUTP nick end labeling) staining, also called the TUNEL Assay, detects the DNA breaks formed when DNA fragmentation occurs in the last phase of apoptosis [182]. TdT can label blunt ends of double-stranded DNA breaks as it attaches to deoxynucleotides to the 3′-hydroxyl terminus of DNA breaks. The nucleotides attached by TdT are stained with a fluorescent dye. Tunnel assay is an alternative assay to agarose gel electrophoreses to analyze the formation of DNA fragments during apoptosis. Teraoka et al., used this assay to access apoptotic cells by counting TUNEL-positive cells [156].
8. Immunoblotting/Western blotting: Immunoblotting, or western blotting, is used to identify changes in protein expression following treatment. In this assay, protein expression is sampled at different time-points following X-ray irradiation to determine how pretreatment with NPs enhances the radiation sensitivity. This is performed both in the presence and absence of NPs [123]. This assay uses protein expression levels in different time points after radiation. Primary antibodies can be selected according to selected interest such as apoptosis, DNA damage and repair and oxidative stress.
9. Immunocyto/histochemistry for Cellular Senescence detection: There is a number of senescence biomarkers. The most common markers are SA-β-Gal, senescence-associated secretory phenotype (SASP) [183], cell cycle inhibitors p16Ink4a and p21Cip1 [184], and lipofuscin [185]. Oxidative stress is one of the possible reasons leading to senescence. Irradiation alone can lead to increased senescent phenotype [186]. Since ROS production can be responsible for NP-induced radiosensitization, it is essential to also study whether or not irradiated cells incubated with NPs lead to increased senescence compared to the irradiated alone. Until now there are only few groups who address senescence [173,187].

## 5. Clinicals Trials

### 5.1. The Use of NPs in Radiotherapy

Despite all the above challenges and the multiscale approaches for an effective NP-boosted radiation therapy (RT), some clinical trials were already conducted with NPs. Among the various types of the metallic NPs, Gadolinium based NPs are demonstrated as a very promising agents for RT treatment. AGuIX Gadolinium-based NPs were passed to phase II as radiosensitizers with Stereotactic Magnetic Resonance-guided Adaptive Radiation Therapy (SBRT) for the treatment of lung and pancreatic cancer ((NCT number): NCT04789486). Radiation by proton therapy associated to AGulX NPs injection is also on phase II for patients with tumor of the cephalo-spino-iliosacral axis, particularly base of the skull, pharyngeal wall, parapharyngeal and retropharyngeal lymph nodes etc. ((NCT number): NCT04784221). Hafnium oxide (hafnia, HfO_2_) are also shown to be a promising sensitizer for radiation therapy as well as x-ray contrast agents [188,189]. Nanobiotix (http://www.nanobiotix.com/_en/ (accessed on 28 April 2021)) developed NBTXR3, which are functionalized HfO_2_ NPs. These NPs are 50 nm sized crystalline nanoparticles, with a bearing negative surface charge. Their design was for direct local intratumoral injection and subsequent radiosensitization [67,190]. Until recently, several clinical trials were carried out using NBTXR3 crystalline nanoparticles-RT combination. A phase I trial with the use of NBTXR3 in sarcoma patients started in 2011 and completed in 2015 ((NCT number): NCT01433068) [191]. More phase I/II trials and one phase II/III trial followed which included participants with various cancer types such as head and neck cancer (squamous cell carcinoma) ((NCT number): NCT01946867), rectal cancer, hepatocellular carcinoma (liver cancer) ((NCT number): NCT02721056), prostate cancer ((NCT number): NCT02805894), and adult soft tissue sarcoma ((NCT number): NCT02379845). All the previously mentioned clinical trials are summarized below in Table 1.

### 5.2. Limitations and Challenges in Using Nanoparticles in Clinic

To introduce nanoparticles into clinical use, we have to take into consideration several biological and technical limitations. Even though each individual type of NP will provide different challenges, most of nanomedicines will face the same limitations. Here, we will summarize very briefly these limitations. Biological challenges include nanotoxicity, as well as biodistribution or controlling passage of NPs across biological barriers and into specific targeted cells or tissues. These are directly connected to surface functionalization and particle size. Specific surfactants, coating (e.g., PEG), or the use of lipids, peptides, and proteins (e.g., human serum albumin) can enhance biocompatibility and are acceptable for clinical use [192,193]. Some of these types of functionalized nanoparticles may also provide additional advantages for internalization and crossing of biological barriers [194]. Another major issue is the crossing from preclinical (in vivo experiments) to clinical use. Due to the heterogeneity of humans and animal species the optimizations in animal models (e.g., small animals), may not work the same in humans. Added to this, is the fact that unlike animals, the biodistribution (in organ and tissue level) of nanoparticles in humans cannot be easily determined. The only way to monitor nanoparticle fate and biodistribution (e.g., penetration through tumor) in humans is through biopsies [195] or imaging-based nanoparticles [196,197]. However, the need to determine the biodistribution of NPs can be reduced significantly, by using tumor targeted functionalized NPs in lower concentrations. In that case, the toxicity and the effectiveness of treatment could be also monitored by other means than biopsies, enabling this way the incorporation of NPs into cancer treatment.

## 6. Discussion

**Figure 3:**
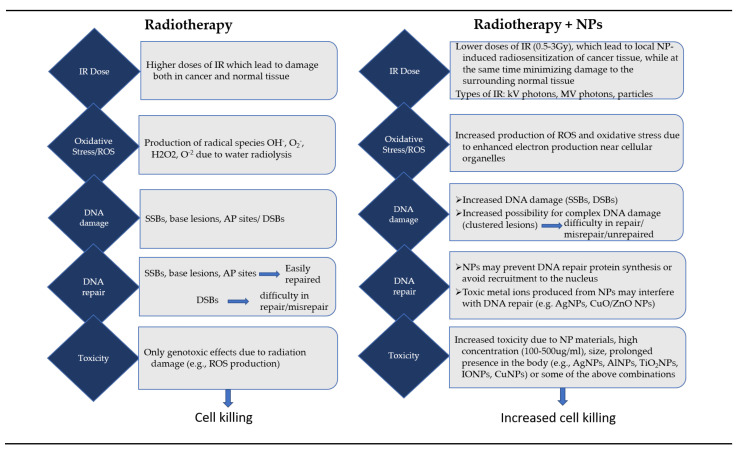
A summary of key point differences between RT and combined NP radiotherapy.

To summarize all of the above results and for better understanding of the key differences between RT and combined RT with NPs, we provide below Figure 3.

Apart from the material of the NPs and the use of a specific radiation source, the choice of the delivery route and the interactions of NPs with the biological systems (for both tumor and normal cells), which depends on the physical and chemical characteristics of NPs [3], contribute significantly to the level of radiosensitization. However, since we discuss about the clinical application of NPs, it would be important here to address also the matter of NP elimination from the body after their use.

The use of nanomaterials in clinical research radiotherapy is troubleshooting. The fate of nanoparticles after the delivery inside the human body raise concerns about their chronic accumulation and consequently about the patient safety. This is one of the main reasons why the application of NPs as radiosensitizers is very limited and very rare in clinical trials. The United States Food and Drug Administration (FDA) requires nanoparticles that can be metabolized and excreted from the body once they have fulfilled their purpose. Studies show that nanoparticles injected into the bloodstream of laboratory animals are found in organs including the liver, spleen, heart, and brain. Nanoparticles in the blood can be filtered out by the kidneys and excreted in urine. Those administered intravenously circulate in the blood until they are cleared from circulation and eliminated from the body by two main mechanisms: renal elimination and hepatobiliary elimination [198]. Most nanoparticles with dimensions less ∼5.5 nm undergo renal elimination by the kidneys and leave the body via the urine [199]. Based on this knowledge, many strategies for tuning nanoparticle renal elimination were since developed using nanoparticle size, core density, surface charge, and surface chemistry. Even if larger sizes of NPs ranging from 20 to 60 nm provide better cellular uptake, smaller NPs still accumulate in tumors due to the EPR effect and because of the smaller size diffuse further into the tumor tissue and afterwards they can be eliminated from the blood stream [200,201]. Biodegradable NPs that are larger than 5 nm can be disassembled, broken down, or metabolized and may return to the systemic circulation [202,203]. On the other hand, most of the larger nonbiodegradable NPs become retained long-term in Kupffer cells [204], otherwise they could undergo hepatobiliary elimination. Even then, however, they must undergo certain barriers to enter the bile ducts and be removed. Eventually, the nanoparticles enter the intestines and are removed from the body via feces. Sadauskas et al., reported that liver clearance was about 9% in 6 months [205], and eventually this whole-body retention will be a problem for FDA approval. In previous sections we discussed local intratumoral injection. This is considered to be invasive depending on the tumor site, and there is relatively rapid clearance of the drug-conjugated NPs from the tumor volume into systemic circulation, which could lead to drug toxicity in surrounding tissues [206].

Among the metallic nanoparticles, gold is a very promising candidate and even though it is expensive, it could be used selectively for cancer radiotherapy in small amounts. A problem though with gold, is that is causes skin discoloration [207]. Nanoparticles <6 nm in a biodegradable matrix were developed for better clearance to extend blood life and reach the kidneys. However, during the long circulation they might be taken up by macrophages and get trapped in lysosomes [208]. High-Z NP x-ray dose enhancement is better with kV photons than megavoltage, but MV instruments are the ones currently used clinically due to better penetration to deep located tumors. In addition, experiments show good dose enhancement with less than ~2% Au by weight, even with MV photons [209].

Even if MV photons are more abundant in cancer therapy, kV photons come again into play with microbeams or minibeams [210]. kV machines are less expensive, require less shielding, and could make NP-induced cancer radiotherapy available to most countries. Moreover, based on all the above things which are discussed in this paper, current radiation therapy modalities starting from low-LET (X-rays and electrons) up to protons and carbons (high-LET) offer unique tools for clinicians to achieve the ultimate goal, i.e., tumor control and minimization of normal tissue toxicity. Of course, each type of radiation type and treatment carries its own constraints and limitations. The use of protons and carbons as the main type of high-LET particles used in RT, come with the advantage of higher dose delivery to the tumor area and minimum impact to the surrounding tissues compared to low-LET RT treatments [211,212]. Nowadays, new very high-dose rate approaches evolved, delivering exciting opportunities for cancer treatment called FLASH-RT. Based on cellular and higher mammals’ studies, the suggested dose rates of up to 100 Gy/s, using protons or X-ray/electrons synchrotron beams, will minimize the radiation toxicity. However, FLASH-RT was not yet tested on human patients [213,214].

## 7. Conclusions

The radiosensitizing and synergistic effects of metallic NPs are a combination of physical and chemical dose enhancements properties, followed by a biological phase. The magnitude of the dose enhancement depends on many physical, chemical, and biological parameters, e.g., the quantity and quality of the dose of the IR, the size, shape, structure, coating, functionalization, concentration, of the metallic-based NPs, as well as the availability of oxygen, the cell uptake, and the intracellular distribution. This review also tries to present the complexity of the damage (e.g., DNA damage, oxidative stress) either from direct or indirect effect. The emission of electron and the consecutive ROS clusters favor this induction of complex damage. There is still need for optimized protocols in radiotherapy treatment for a translation to the clinical practice. The entrance of the metallic-based NPs in clinical practice to improve the effect of IR therapy demands the consideration of different effects/mechanisms. This includes the physical and chemical dose enhancement effects, as well as the biological effects, including cellular uptake, localization, toxicity, oxidative stress, effects on the cell cycle, DNA damage, apoptosis, autophagy, and senescence. NPs or NP-based structures clinically used as radiosensitizers should ideally be designed to have sensitivity to the tumor environment and specific stimuli, such as temperature, pH, magnetic field, radiation, etc. Therefore, delivery techniques/routes of NPs for RT applications also play a very important role in tumor sensitization, resulting in radiotherapeutic dose enhancement. Thus, integrated strategies need to be developed for an effective metallic nanoparticles radiosensitization. In this paper, we summarized all the recent clinical trials based on metallic NP radiotherapy along with their limitations in terms of clinical use. For the addition of NPs in preclinical, but most importantly, in clinical research, it is now becoming clearer that in the future the construction of NPs should be focused on targeting tumor sites specifically. Conventional radiotherapy alone was tremendously improved. If we could manage to use all the knowledge about the material, size, shape and functionalization, we would be able, at least in certain cancer types, to introduce NPs or drug/NP-based structure in clinical trials and reduce the cytotoxic effects on the surrounding tissues.

## Figures and Tables

**Table 1:** Combined nanoparticle-radiation therapies in cancer patients. Table includes currently undergoing or completed clinical trials (not yet recruiting, recruiting, active, completed).

Name (Sponsor)	Particle Type/Drug	Application/Indication	Patients	Type of IR/Dose	ClinicalTrials.gov Identifier (Phase)	Status
AGuIX (University Hospital, Grenoble)	Gadolinium-based nanoparticles	Target tissue: Multiple brain metastases Application: Sequentially rising dose levels of AGuIX NPs were injected in combination with whole brain radiation therapy (Ph I). Based on adapted dose, AGuIX NPs are injected in combination with whole brain radiation therapy (Ph II).	15 100	X-rays/30Gy (3Gy/session)	NCT02820454 (Ph I) [68] NCT03818386 (Ph II)	Completed recruiting
AGuIX (University Hospital, Grenoble)	Gadolinium-based nanoparticles	Target tissue: Pancreatic and lung cancer Application: AGuIX NPs are injected in combination with MR-guided stereotactic body radiation therapy (SBRT)	100	X-rays/-	NCT04789486 (Ph I/II)	recruiting
AGulX (Centre Francois Baclesse)	Polysiloxane and Gadolinium-based nanoparticles	Target tissue: Relapsing tumors of the cephalo-spino-iliosacral axis particularly base of the skull, pharyngeal wall, parapharyngeal, and retropharyngeal lymph nodes, etc. Application: AGuIX NPs are injected in combination with photon therapy	46	Photon therapy/-	NCT04784221 (Ph II)	Not yet recruiting
NBTXR3 PEP503 (Nanobiotix)	Hafnium oxide-based nanoparticles	Target tissue: locally advanced soft-tissue sarcoma of the Extremity and Trunk Wall Application: NPs were stimulated with external radiation to enhance tumor cell death via electron production	180	External beam radiotherapy/ 50Gy(2Gy/fraction)	NCT02379845 (Ph II/III) [66]	completed
NBTXR3 PEP503 (Nanobiotix)	Hafnium oxide-based nanoparticles	Target tissue: locally advanced squamous cell carcinoma of the oral Application: NPs are stimulated with external radiation to enhance tumor cell death via electron production	48	Intensity-Modulated Radiation Therapy /70Gy (2Gy/fraction)	NCT01946867 (Ph I)	recruiting
NBTXR3 PEP503 (Nanobiotix)	Hafnium oxide- based nanoparticles/PD-I inhibitor	Target tissue: advanced cancer types Application: NPs are stimulated with external radiation to enhance tumor cell death via electron production in combination with immunotherapy	60	Radiotherapy/-	NCT03589339 (Ph I)	recruiting
NBTXR3 PEP503 (Nanobiotix)	Hafnium oxide-based nanoparticles	Target tissue: liver cancers Application: NPs are stimulated with external radiation to enhance tumor cell death via electron production NCT02721056	23 (initial nu. 200)	Stereotactic Body Radiation Therapy/45Gy (15Gy/fraction) or 50Gy (5Gy/fraction)	NCT02721056 (Ph I), Ph II will be designed independently	terminated
NBTXR3 PEP503 (Nanobiotix)	Hafnium oxide-based nanoparticles	Target tissue: Prostate Adenocarcinoma Application: NPs are stimulated with external radiation with and without brachytherapy to enhance tumor cell death via electron production.	5(initial nu. 96)	External Beam Radiation Therapy/45Gy (1.8Gy/ fraction) or Brachytherapy Boost/15Gy + EBRT 45Gy (1.8/fraction)	NCT02805894 (PhI/II)	terminated
SPION	Iron oxide Nanoparticles/ ferumoxytol	Target tissue: Primary and metastatic hepatic cancers Application: MR-Linac imaging with SPION in combination with radiotherapy to increase detection and efficacy.	25	Stereotactic Body Radiotherapy (LINAC)/-	NCT04682847	recruiting

## Data Availability

The data presented in this study is contained within this article.
